# Workshop on RanBP2/Nup358 and acute necrotizing encephalopathy

**DOI:** 10.1080/19491034.2022.2069071

**Published:** 2022-04-29

**Authors:** Alexander F. Palazzo, Jomon Joseph, Ming Lim, Kiran T. Thakur

**Affiliations:** a Department of Biochemistry, University of Toronto, Toronto; b National Centre for Cell Science, S.P. Pune University Campus, Pune, India; c Children’s Neurosciences, Evelina London Children’s Hospital, and the Department of Women and Children’s Health, King’s College London, London, UK; d Department of Neurology, Vagelos College of Physicians and Surgeons, Columbia University Irving Medical Center, and the New York Presbyterian Hospital, New York

**Keywords:** Nuclear pore complex, influenza, cytokine storm, genetic disease, RanBP2, Nup358

## Abstract

Dominant missense mutations in RanBP2/Nup358 cause Acute Necrotizing Encephalopathy (ANE), a pediatric disease where seemingly healthy individuals develop a cytokine storm that is restricted to the central nervous system in response to viral infection. Untreated, this condition leads to seizures, coma, long-term neurological damage and a high rate of mortality. The exact mechanism by which RanBP2 mutations contribute to the development of ANE remains elusive. In November 2021, a number of clinicians and basic scientists presented their work on this disease and on the interactions between RanBP2/Nup358, viral infections, the innate immune response and other cellular processes.

## Introduction

**Figure 1.**
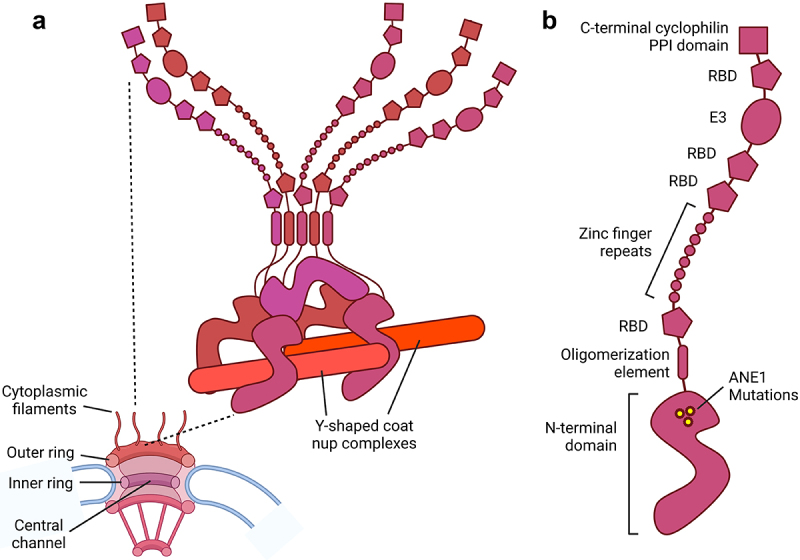
**RanBP2/Nup358, a giant nuclear pore-associated protein**. (a) RanBP2/Nup358 is one of the major components of the cytoplasmic filaments of the nuclear pore complex, which has an eightfold symmetry, with each symmetrical unit typically referred to as a ‘spoke’. The eight filaments sit on top of the outer ring, which is composed of sixteen copies of the Y-shaped coat nup complex. Five copies of RanBP2/Nup358 are found at each of the eight spokes of the pore, for a total of 40 copies per pore. A pair of RanBP2/Nup358 molecules clamp to one copy of the Y-shaped coat nup complex by their N-terminal domains. This structure is then duplicated to give two pairs of RanBP2/Nup358 at each spoke. The fifth copy of RanBP2/Nup358 sits on top of the other four and these are held together with the aid of the oligomerization element. (b) RanBP2/Nup358 has several domains, including the N-terminal domain which not only attaches to the pore, but is where the ANE1 mutations reside. The five copies are held together by an oligomerization element. RanBP2/Nup358 also contains four Ran Binding Domains (RBDs), eight zinc fingers, a SUMO E3 ligase domain and a C-terminal cyclophilin peptidyl *cis-trans* prolyl isomerase (PPI) domain. This model of RanBP2/Nup358 was adapted from Bley et al., 2022 and designed on BioRender.com.

In 2009, Derek Neilson and colleagues published a seminal paper where they reported that three separate dominant missense mutations in the *RANBP2* gene were associated with Acute Necrotizing Encephalopathy (ANE), a pediatric condition where otherwise normal individuals develop a cytokine storm localized primarily to the central nervous system in response to viral infection [1]. Not many inherited genetic diseases are caused by dominant mutations, in part because they can affect all carriers, although many severe neurological disorders can be caused by *de novo* dominant mutations. In the case of mutations in *RANBP2*, they are only 40% penetrant, at least according to the initial reports, and thus these alterations are often passed down by unaffected carriers. How these mutations cause ANE remains unclear [for excellent recent reviews on this topic see 2, and 3]. This gene encodes the RanBP2/Nup358 protein, which is ubiquitously expressed and is part of the cytoplasmic filaments that are present on the cytoplasmic face of the nuclear pore complex [4,5] (Figure 1a). Critically, RanBP2/Nup358 has an E3 SUMO-ligase domain (Figure 1b) which can promote the covalent attachment of the small ubiquitin-like modifying (SUMO) protein to the lysine side chains of other proteins [6,7], and this likely contributes greatly to its cellular functions.

Although mutations in the *RANBP2* gene are associated with ANE (this genetic form is sometimes referred to as ANE1), there are individuals with ANE who do not have known disease-causing variants in this gene. Moreover, there appear to be other genetic determinants that may explain why RANBP2 mutations are only 40% penetrant; however, this remains unclear.

To shed some light on this disease, numerous clinicians and basic researchers convened from 10 to 12 November online to share their findings and discuss issues that need to be addressed to make progress in understanding the underlying etiology of this devastating condition. The topics discussed not only touched upon the role of RanBP2/Nup358 in cell function, viral infection and the innate immune response, but also the contribution of other nucleoporins to neurodegeneration diseases, the role of SUMO in regulating cell physiology, and the use of model systems to investigate interactions between airway epithelial cells and viral infection. The meeting was attended by 114 participants from 20 countries and included not only researchers but also primary care physicians and ANE family members.

On the first day, Kim Smith from ANE International gave introductory remarks about the formation of the group, which includes close to 200 families worldwide. ANE International was founded in 2016, and its mission is to support families, educate on the disease, and raise awareness. When they were formed, ANE was not listed on many rare disease databases. They now have a website (https://aneinternational.org/) and social media presence on both Facebook and Twitter. They are in constant contact with the research community and were instrumental in organizing this workshop.

## Clinical aspects of ANE

The clinical introduction was covered by four talks comprising an introduction to the clinical spectrum of children presenting with virus-associated encephalopathy (Ming Lim, Evelina London Children’s Hospital, UK), the investigations of children with encephalopathy with a focus on the para-infectious inflammatory encephalitides (Russell Dale, Children’s Hospital at Westmead, University of Sydney, Australia), the genetically determined form of acute necrotizing encephalopathy (ANE) with *RANBP2* mutations (Derek Neilson, Phoenix Children’s Hospital, USA) and infectious triggers including emerging data on its association with COVID (Kiran Thakur, Columbia University Irving Medical Center, USA).

A ‘neurological deterioration’ in a child presents a major worry to the family and frequently presents a diagnostic challenge to the clinician. The presence of preceding or concurrent infection and clinical features of encephalopathy such as altered consciousness that persists for longer than 24 hours, lethargy, irritability, or a change in personality and behavior [8] raises the possibility of an infection and/or immune-mediated process affecting the brain, alongside a large differential diagnosis of childhood encephalopathy [9].

Within the children presenting with virus associated encephalopathy, distinct clinical and radiological syndromes have been reported ranging from the milder encephalitis/encephalopathy with a reversible splenial lesion (MERS), through to acute necrotizing encephalopathy (ANE), acute encephalopathy with biphasic seizures and restricted diffusion (AESD) and hemorrhagic shock and encephalopathy syndrome (HSES), as reviewed by others [10]. Whether these syndromes represent simply a spectrum disorder or possibly involve distinct pathomechanisms is currently being evaluated.

**Figure 2.**
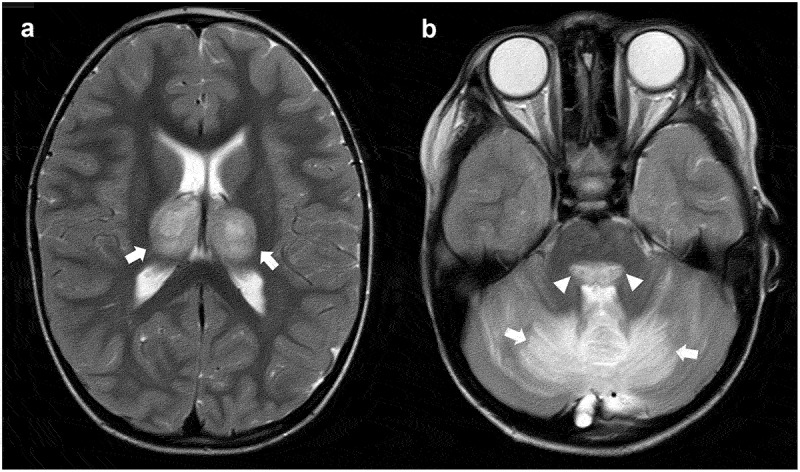
**Magnetic resonance imaging in a 3 year old presenting with a viral prodrome followed by rapid neurological deterioration**. T2-weighted axial images demonstrating the characteristic thalamic lesions (Panel A, arrows), alongside more widespread changes in brainstem (Panel B, arrow heads) and cerebellum (Panel B, arrows) seen in acute necrotizing encephalopathy.

ANE has been identified in the context of many infections including Influenza A and B, Parainfluenza, Varicella, HHV6, Parvovirus B19, Enterovirus, Reovirus, Rotavirus, Measles, Mycoplasma, Diphtheria, and Streptococcus. ANE is implicated in up to 10% of influenza-associated encephalopathies, particularly during the H1N1 swine flu pandemic [11]. Neuroimaging is key in identifying the hallmark thalamic changes often including multifocal lesions in the brainstem, cerebral periventricular white matter, and cerebellum [12]. The changes are often symmetric (Figure 2) and may additionally be of restricted diffusion. Cerebrospinal fluid (CSF) evaluation often reveals a raised protein level in the absence of pleocytosis. Although PCR for some viruses is positive in CSF in some reports, there are no signs of encephalitis on autopsy and minimal brain inflammation in relation to the marked parenchymal abnormality. Inflammatory factors are implicated in ANE, with elevations in serum pro-inflammatory cytokines [13] being reported, but the clinical utility of these observations in diagnosis and management remains limited. ANE has been described in predominantly adults with SARS-CoV-2 (COVID-19) infection 14–18]. Of note, there was variation in the severity of COVID-19 infection, and early manifestations are similar to other ANE cases although they are not hyperacute .

Children with mild ANE may only have febrile illness and concurrent respiratory or gastrointestinal tract infection, but children with more severe ANE, however, deteriorate rapidly in the first days of the febrile illness and need admission to the intensive care unit (ICU) at the hospital, requiring neuroprotection and treatment of seizures. Given the presumed inflammatory nature of ANE, first-line immunotherapy with steroids, intravenous immunoglobulin and plasma exchange are often employed as in other neuro-inflammatory disorders, with one study suggesting that steroid treatment given within the first day of presentation is associated with better outcomes [19]. Newer strategies such as with interleukin-6 receptor blockage have been employed in a handful of more severe cases with apparent benefit [20]. The recovery following an episode of ANE is very varied, with some making a near full neurological recovery and many children left with profound disability [21,22] including a mortality rate of up to 30%.

In most children, ANE is monophasic. However, in some children, recurrent episodes of ANE occur due to an inherited genetic predisposition. The most common gene where mutations are identified is *RANBP2* [1]. In a recently published Canadian cohort, 7/20 (23%) ANE cases were *RANBP2* mutation positive [23], although most cases from Asian cohorts appear to be mutation negative (personal communication M. Lim and R. Dale). As described above, this gene encodes a nuclear pore protein, RanBP2/Nup358, and has been implicated in many cellular functions (see below). As such, the vastly increasing knowledge on this protein may provide new therapeutic insights into ANE and the spectrum of virus-associated encephalopathy, which currently remains clinically and radiologically indistinguishable from the genetic form. The recurrence risk and also familial risk require family-specific management that includes influenza vaccinations and very low threshold to initiate investigations and expeditious immune treatment with each intercurrent illness.

## Structural studies on RanBP2/Nup358 and the nuclear pore complex

The nuclear pore complex is the sole bidirectional gateway for nucleocytoplasmic transport. Despite recent progress in elucidating the core architecture of the pore, the structure of the filaments, which have been shown to be essential for mRNA export and a hotspot for nucleoporin-associated diseases, has remained elusive. André Hoelz (CalTech, USA) presented recent work from his lab that begins to piece together how the filament proteins, including RanBP2/Nup358, are arranged [24]. In particular, they solved the crystal structures of all the RanBP2/Nup358 domains including the N-terminal alpha-helical solenoid region (amino acids 1–752), which harbors the ANE mutations (Figure 1b). They demonstrated that this region is composed of three distinct alpha-helical solenoids that interact in a novel manner, adopting a unique overall S-shaped architecture with a propensity to form domain-swapped homo-dimers. They found that amino acid sites that are altered by ANE mutations all reside in one of these solenoids where they all face the interior of the fold. His group then found that all these mutations reduce the thermostability of this domain. This region is followed by an ~ 50-residue linker and an oligomerization alpha-helix that forms homo-tetramers/pentamers in solution. In agreement with this, the Hoelz lab found that CryoEM-derived models of the nuclear pore complex can accommodate five copies of RanBP2/Nup358 for each of the eight spokes on the pore (Figure 1a). They then found that the N-terminal domain binds to single and double stranded RNAs, as suggested by previous studies [25]. In collaboration with the Dasso lab, they engineered human tissue culture cells where the endogenous RanBP2/Nup358 contains an auxin-induced degron and where all of the protein can be eliminated within 3 hours, at which point the cells displayed defects in the translation of many reporter mRNAs, in agreement with previous findings [26].

Other structural knowledge about how nucleoporins interact with one another was presented by Radha Chauhan (National Centre for Cell Science, Pune, India). Her studies focused on Nup62, which is present both in the cytoplasmic filaments and in the central channel of the nuclear pore (Figure 1a). She described how a point mutation in Nup62 (Q391P) causes Infantile Bilateral Striatal Necrosis (IBSN), a syndrome that has overlapping features with ANE, but is considered distinct. Nup62 has an extended unfolded N-terminal and a structured C-terminal region where the IBSN mutation is found. This region forms a trimeric coiled-coil complex either as a homo-trimer or in various hetero-trimeric configurations with two other nucleoporins (Nup54 and Nup58) [27]. These three proteins form the central transport channel complex. Nup93 interacts with the Nup62-Nup54-Nup58 coiled-coil structure and alters its overall shape [28]. In more recent unpublished work, her lab has characterized how Nup62 also interacts with Nup88 and Nup214, two nucleoporins that sit on the cytoplasmic face of the nuclear pore and with RanBP2/Nup358 form the cytoplasmic filaments that emanate from the pore. These three nucleoporins also appear to form a coiled-coil trimer. This reconstitution was greatly facilitated by a machine learning-based algorithm (Co-evolution, Random Forest, and Network Analysis, namely, CoRNeA) that predicts protein-protein interaction interfaces [29]. Chauhan then pointed out that Nup62 has a residency time of approximately 13 hours in the pore [30] and suggested that it may alter its interaction partners over this time course.

One of the main interactors of human RanBP2/Nup358 is RanGAP1, and the significance of this interaction was discussed by Mary Dasso (National Institutes for Health, USA). Although it is known that this binding anchors RanGAP1 to the cytoplasmic face of the nuclear pore in vertebrates, the reasons for this remain unclear. RanGAP1 activates Ran GTPase activity and helps to maintain the RanGTP-gradient where nucleoplasmic Ran is GTP bound, while cytoplasmic Ran is GDP-bound. This RanGTP gradient is essential for promoting the nuclear-cytoplasmic trafficking of many macromolecules. In vertebrates, RanBP2/Nup358 has an E3 SUMO-ligase that promotes the SUMOylation of RanGAP1. This modification allows RanGAP1 to bind to the pore in mammalian cells [31,32]. Attachment to the nuclear pore is mediated by the E3 ligase region, which forms a complex with SUMO-RanGAP1 and Ubc9, the E2 SUMO-ligase [33]. RanBP2 and RanGAP1 also play a role in mitosis, where they promote the proper attachment of kinetochores to the spindle microtubules [34,35]. When the Dasso lab created a cell line where the SUMOylated lysine in RanGAP1 was mutated to arginine, which prevents its SUMOylation and its anchoring to the pore, they found no significant changes in the viability of the cells or of the rate of nuclear import or export of proteins [36]. They also did not detect any problems in mitosis. These observations raised the question of why RanGAP1 associates with the nuclear pore in diverse organisms. To address this, the Dasso lab asked whether the nuclear pore-anchoring of RanGAP1 was required for the development of fruit flies (*Drosophila melanogaster*). Dasso pointed out that in unicellular eukaryotes, such as yeast, RanGAP1 is present diffusely throughout the cytoplasm, but that in many multicellular eukaryotes, including plants and invertebrates, RanGAP1 is anchored to the outer nuclear envelope and/or the nuclear pore. Although it is known how RanGAP1 localizes to the nuclear envelope in plants [37], it remained unclear how it was localized to the nuclear pore in flies. Dasso’s group found that it was anchored by the fly RanBP2/Nup358, but via a divergent mechanism. The fly RanBP2/Nup358 lacks an E3 domain and instead binds to RanGAP1 by a 23 amino acid sequence not found in vertebrates [36]. Flies that are engineered such that their RanBP2/Nup358 lacks this region fail to anchor RanGAP1 to the nuclear pores and show developmental defects, although these arise late in development during pupation, indicating that most basic cellular functions appear to be operational [36]. Dasso speculates that the anchoring of RanGAP1 to the nuclear pore may be required for specialized nuclear transport events or signaling pathways at key points during development, but the exact details remain to be elucidated.

In the last few years, there have been a number of studies linking nuclear pore dysfunction with neuropathology. This topic was explored in a talk by Jefferey Rothstein (John Hopkins University, USA). He described his lab’s work in investigating how a hexanucleotide (GGGGCC) repeat expansion in *C9orf72* promotes Amyotrophic lateral sclerosis (ALS). Key in the development of this disease is the relocalization of TDP43, a nuclear protein, to the cytoplasm where it eventually aggregates. The Rothstein group had seen that neuronal cells expressing *C9orf72* with repeat expansions had aggregates of RanGAP1 in the cytosol and a defect in nuclear trafficking [38]. In more recent work, they demonstrated that these cells also had defective pores that lacked eight different nucleoporins [39]. Surprisingly, the defect in nuclear trafficking in these cells could be rescued by overexpressing POM121, one of the few nucleoporins that is membrane bound and anchors the inner ring of the nuclear pore to the nuclear envelope (Figure 1a). Since depletion of POM121 also leads to a selective loss of the same eight nucleoporins from pores, it appears to be a key component in regulating the assembly of these particular pore components. In collaboration with the Lusk lab (Yale School of Medicine), the Rothstein group has now linked this restructuring of the pore to the activation of the nuclear envelope quality control pathway, which had been previously defined in yeast and involves the ESCRT III complex and Chm7 [40,41]. This pathway is activated when the nuclear envelope ruptures and Chm7, which is normally excluded from the cytoplasm, accesses the inner membrane protein Heh1 (LEM2 in humans). This binding event then promotes nuclear envelope resealing by the ESCRT III complex. The Rothstein group, in collaboration with the Lusk lab, determined that the human ortholog of Chm7, CHMP7, is recruited to the nuclei of cells with *C9orf72* repeat expansions and ALS patient cells before their nuclear pores lose nucleoporin components [42]. This observation suggests that ALS patient cells experience leakage between the cytosol and nuclear compartments. They then found that the overexpression of nuclear targeted CHMP7 promotes the partial disassembly of the pores and the nuclear export of TDP43. Conversely, depletion of CHMP7 restores the nuclear pore complex and nuclear localization of TDP43. These results strongly indicate that the inappropriate activation of the nuclear envelope quality control pathway may be a key trigger of ALS and perhaps other neurological diseases.

## Interactions between RanBP2/Nup358 and other nuclear components with viruses

Many viruses use nuclear pores to gain access to the nucleoplasm, which is essential for viral production [43,44]. Nelly Panté (University of British Columbia, Canada) gave an overview of her lab’s work, where they uncovered different strategies used by viruses to enter the nucleus. Two significant human pathogens, influenza A virus and hepatitis B virus (HBV), cross the nuclear pore complex hijacking the nuclear import machinery, but using fascinating new mechanisms to deliver their genome into the nucleus. Influenza A virus uses two non-classical nuclear localization sequences (NLSs) that function in synergy by interacting with the minor and major NLS-binding pockets of importin-α to deliver the viral genome into the nucleus [45]. The capsid of HBV contains classical NLSs and crosses the NPC in an importin-α and -β-dependent manner. The HBV capsid then binds directly to Nup153 at the nuclear basket, which triggers disassembly of the capsid and nuclear entry of the viral genome [46]. While some viruses hijack the nuclear import machinery, others use NLS/importin-independent mechanisms to deliver their genome into the nucleus. For example, baculovirus enters the nucleus using propulsive force of actin polymerization [47–50] and parvovirus transient disrupts the nuclear envelope and the underlying nuclear lamina and enters the nucleus through the resulting breaks [51,52]. These findings indicate that the well-characterized cellular mechanism to enter the nucleus may not be the only avenue for nuclear import.

Interactions between viruses and nuclear pore components can be both advantageous and deleterious to the virus. Natalie Arhel (Université de Montpellier, France) spoke about the interplay between the Human Immunodeficiency Virus 1 (HIV-1), nuclear pore complexes and the innate immune response. HIV uses its capsid to dock to nuclear pore complexes through direct interactions with RanBP2/Nup358 [53–55], while other components of the nuclear pore complex machinery play key roles in HIV replication. This includes the nuclear transport receptor, TRN-1, which ferries HIV capsid into the nucleus and uncoats viral complexes [56], and other nucleoporins, which direct HIV integration into the genome [57,58]. In some cases, nucleoporins may even be co-opted by viruses to evade the innate immune response. For example, RanBP2/Nup358 SUMOylation activity suppresses the innate immune sensing of viral cDNA in the nucleus [59]. In other cases, nuclear pore components turn on antiviral factors. For example, RanBP2/Nup358-dependent SUMOylation of TRIM5-alpha activates its ability to uncoat viruses and promote abortive infection [60,61]. In unpublished data, the Arhel lab has observed that RanBP2-depletion favors the production of pro-inflammatory cytokines by human peripheral blood mononuclear cells, in particular by CD8+ lymphocytes, CD4+ lymphocytes and natural killer cells. This tug of war between viruses and innate immunity for the nuclear pore complex suggests that it could be a site for virus host-cell co-evolution. This is illustrated by a publication from Sara Sawyer’s group, which showed that the Simian Immunodeficiency Virus-to-HIV spillover required viral adaptation to RanBP2, suggesting that nuclear pore complexes drive spillover of viruses to new host species [62].

Since viruses like influenza must interact with nuclear components as part of their replication cycle, these interactions can be therapeutically targeted to develop new antiviral compounds. Within this context, Beatriz Fontoura (University of Texas, Southwestern, USA) spoke about how her group was developing new compounds that target key interactions between viral proteins and host machinery in the nucleoplasm, to disrupt influenza viral replication [63,64].

One of the key aspects in furthering our understanding of how respiratory viruses interact with host cells, and how this triggers various immune responses, is to develop new tissue culture models of airway epithelial cells. Theo Moraes (SickKids, University of Toronto, Canada) described how his group has leveraged primary human airway epithelial cell cultures to study these interactions. His lab collects nasal swabs from patients to generate air liquid interface cultures that allow for a more complete functional characterization of epithelial cells. This system offers a model to study the consequences of perturbations in RanBP2/Nup358 in the context of viral infection. It has been used by his lab to study cystic fibrosis patient cells and how these respond to gene therapy and small-molecule corrector compounds [65–68]. This system requires technical expertise, is relatively more expensive than traditional cell culture and is less amenable to genetic manipulation. Nevertheless, these cells are the primary target for respiratory viruses and produce cytokines, which are key regulators of inflammation.

## Biology of SUMOylation

As discussed above, RanBP2/Nup358 is one of the major E3 SUMO-ligases in human cells. SUMO, is itself a small ubiquitin-like peptide that is found throughout eukaryotes. Humans have 4 SUMO proteins, namely, SUMO1 through SUMO4. Michael Matunis (John Hopkins University, USA) spoke about his lab’s recent investigations on the role of one of these SUMO proteins. His group developed SUMO2 knockout human cell lines for the analysis of SUMO2 paralog-specific functions [69]. High-throughput RNA sequencing of the SUMO2 knockout cells revealed changes in expression for over 4,000 genes, including *histone* genes and genes coding for type I interferon molecules. The Matunis group observed that the changes in *histone* gene expression resulted from alternative pre-mRNA processing and polyadenylation of *histone* mRNAs and not from changes in transcription. *Histone* pre-mRNAs are alternatively processed and polyadenylated in a number of disease contexts, including the type I interferonopathy, Aicardi Goutieres Syndrome (AGS) [70]. Matunis hypothesizes that inhibition of SUMO2 modification activates type I interferon signaling as an indirect result of alternative *histone* pre-mRNA processing and polyadenylation, as observed in AGS.

SUMO appears to have several roles in regulating cellular processes near the nuclear envelope and at the nuclear pore. The inner nuclear membrane and nuclear pore complexes bind to chromatin and thus contribute to the spatial organization of the genome and epigenetic programs important for gene expression. During mitosis, chromatin-nuclear envelope interactions are lost and then formed again as sister chromosomes segregate to post-mitotic nuclei. Within this context, Richard Wozniak (University of Alberta, Canada) presented work from his lab which explored the relationship between SUMOylation, phosphorylation and remodeling of the nuclear envelope during mitosis in the budding yeast *S. cerevisiae*. His group established that during mitosis, the E3 SUMO-ligase Siz2 relocalized to the nuclear envelope in a phosphorylation-dependent mechanism where Siz2 binds to and SUMOylates the VAP protein Scs2 [71]. The recruitment of Siz2 through Scs2 is further responsible for a wave of SUMOylation along the inner nuclear membrane that supports the assembly and anchorage of subtelomeric chromatin at the nuclear envelope during the later stages of mitosis. These mitotic SUMOylation events also stimulate nuclear envelope membrane production. This process may help to couple the reassociation of chromatin to the nuclear envelope to nuclear envelope membrane expansion pathways required for formation of post-mitotic daughter nuclei.  

SUMO appears to be attached to many proteins in response to cellular stress. Yifan Eva Wang, a PhD student in the Palazzo Lab (University of Toronto, Canada), described her recent work in uncovering novel SUMOylated substrates of RanBP2/Nup358. The Palazzo lab has used CRISPR/Cas9 in human cell lines to modify endogenous RanBP2/Nup358 so that it lacks E3 SUMO-ligase activity [72]. Then, in collaboration with the Raught lab (University Health Network, University of Toronto), these cell lines were used to identify SUMOylated protein substrates of RanBP2/Nup358 by mass spectrometry. Two of the top hits in this screen were G3BP1 and G3BP2, two proteins that have been proposed to form the matrix of stress granules [73]. These structures are cytosolic biomolecular condensates that accumulate translationally repressed mRNAs that form in response to various cell stresses [74]. Furthermore, previous work has suggested that SUMOylation may regulate stress granule assembly and disassembly. Wang presented evidence that RanBP2/Nup358-dependent SUMOylation regulated the dynamics of stress granule disassembly upon the resolution of stress. This new finding is somewhat consistent with previous reports linking SUMOylation and stress granule dynamics [75,76].

## RanBP2/Nup358 and the innate immune response

One of the main features of ANE is the overproduction of cytokines in response to viral infections. This link suggests that RanBP2/Nup358 may directly regulate cytokine production. The association between cytokine protein production and RanBP2/Nup358 was explored by Alex Palazzo (University of Toronto, Canada). His group’s working model is that when mRNAs are synthesized in the nucleus, they are packaged with proteins into messenger ribonucleoprotein (mRNP) complexes, which then undergo a series of maturation events that ultimately dictate how the packaged mRNAs are exported from the nucleus and transported to their correct subcellular destination, how efficiently they are translated into proteins or how they are silenced and destroyed [77]. Previously, his group documented how RanBP2 was required for the efficient translation of mRNAs that encode secretory proteins in human cells. Within this context, it appears that upon the completion of nuclear export, RanBP2/Nup358 directly interacts with parts of the mRNA and promotes the maturation of the mRNP to facilitate translation [26]. In more recent work, his group demonstrated that RanBP2/Nup358 inhibits the translation of mRNAs encoding the Interleukin 6 and Tumor Necrosis Factor alpha cytokine proteins. These particular cytokine mRNAs appear to be packaged into a nuclear mRNP that contains the Argonaute silencing protein, a key component of the RNA-induced silencing complex (RISC). When these mRNPs complete nuclear export, the Argonaute protein must be SUMOylated by RanBP2 to enforce silencing of the mRNA in the cytoplasm [72]. This finding is consistent with previous observations that RanBP2/Nup358-dependent SUMOylation of Argonautes was required for their ability to silence mRNAs [78]. In more recent unpublished work, the Palazzo lab discovered that in the absence of SUMOylation, Argonautes fail to recruit GW182 proteins to the mRNA. GW182 proteins are components of RISC and responsible for recruiting decapping enzymes, deadenylases and translation inhibitory proteins to mRNAs silenced by RISC [79,80]. Interestingly, the Joseph group found that the N-terminal region of RanBP2/Nup358 binds to GW182 and that this is disrupted by ANE mutations [81], although the Palazzo group noted that a mutant form of RanBP2/Nup358 harboring three of the ANE mutations is still able to downregulate the *Interleukin 6* mRNA [72]. Nevertheless, this work provides a potential model of how RanBP2/Nup358 dysregulation could alter cytokine production.

The relationship between RanBP2/Nup358 and the innate immune response was further explored by Qingtang Shen (Fujian Medical University, China). In collaboration with the Palazzo lab, the Shen group has developed a number of human cell lines where the endogenous RanBP2/Nup358 was modified by CRISPR/Cas9 so that it lacks E3 SUMO-ligase activity [72]. Then, in collaboration with the Raught lab (University Health Network, University of Toronto), these cell lines were used to identify SUMOylated protein substrates of RanBP2/Nup358 by mass spectrometry. One of these substrates was STAT1. Shen provided evidence that RanBP2/Nup358-dependent SUMOylation of STAT1 suppresses its ability to activate the transcription of genes with Interferon-Stimulated Response Elements. These results suggest that RanBP2/Nup358-dependent SUMOylation attenuates the innate immune response through repressing STAT1 activity and this may have implications for ANE, which may be the result of a hyperstimulation of these pathways.

## RanBP2/Nup358 in the cytosol

There have been numerous reports that RanBP2/Nup358 can form puncta in the cytosol of many cells [76, 78, 82–84], although the exact nature and function of these remains unclear. Earlier electron microscopic studies revealed that structures similar to the NPCs exist in the cytoplasm as a part of ER, referred to as annulate lamellae. These structures are enriched in a subset of nucleoporins, including RanBP2/Nup358, and have been thought to act as a reserve for NPCs in rapidly dividing cells. However, whether this organelle has additional functions is far from clear.

Interestingly, the cytosolic pool of nucleoporins is also intimately connected with stress granules. As described above, these cytosolic biomolecular condensates accumulate translationally repressed mRNAs [74]. In HeLa cells, RanBP2/Nup358-positive puncta are shown to not only physically associate with arsenite-induced stress granules but also a related structure, P-bodies [78]. This second biomolecular condensate is enriched in RNA decay proteins and mRNAs that are silenced by the RISC pathway [85,86]. It is unclear if annulate lamellae and these biomolecular condensates are different entities, are interchangeable or have similar properties. There have been an increasing number of studies linking the formation of stress granules to alterations in nuclear/cytosolic trafficking [87,88]. Indeed, when stress granules form, they sequester many key nuclear transport receptors and many nucleoporins including RanBP2/Nup358 [76, 87, 88]. It had also been reported that cells depleted of RanBP2/Nup358 had fewer P-bodies [78,89]. Thus, there seems to be a link between RanBP2/Nup358 and RNA-rich biomolecular condensates in the cytosol.

One of the best systems to study annulate lamellae is in early stages of development, as cells undergo many rounds of cell division and thus must rapidly expand the number of NPCs in a relatively short time. This is especially true in fruit fly (*Drosophila melanogaster*) syncytial blastoderm embryos, where cell division is very fast and nuclear pore assembly must be completed during the interphase portion of the cell cycle, which can last as little as 6–12 minutes. This time constraint is a problem as NPC assembly is thought to take at least half an hour. Martin Beck (Max Planck Institute of Biophysics, Frankfurt, Germany) described his lab’s work investigating this very problem. Previously, they found that ALs acted as a reserve of preassembled NPCs that could be directly inserted into growing nuclear envelopes [90]. They reasoned that these nucleoporins must be synthesized and deposited into oocytes by nurse cells, which are germline support cells that synthesize proteins and RNAs that are delivered to the growing oocyte. They found three separate large cytoplasmic condensates composed of different nucleoporins: first, membrane-embedded annulate lamellae that contained a subset of nucleoporins. These were present in the oocyte and accumulated over time. Second, oocyte-specific nucleoporin granules that were not membrane-bound, highly mobile and very heterogeneous in their composition. Third, RanBP2/Nup358 granules, which are large condensates that are assembled in nurse cells and then actively transported along microtubules to the oocyte [83]. By imaging GFP-tagged RanBP2/Nup358 they observed that these granules undergo several maturation steps. First, they are membrane-free and exclude ribosomes. They then accumulate other soluble nucleoporins, often by fusing with other nucleoporin condensates. Then, they surround the AL, all the while excluding ribosomes. In the absence of RanBP2/Nup358, ALs were altered. In the nurse cells, RanBP2/Nup358 granules were surrounded by mRNAs encoding RanBP2/Nup358 and Nup153, and this localization was translation-dependent. In unpublished data, they found that the granules are positive for poly(A)-staining, suggesting that they have other mRNAs. The granules also had RanGAP1, a known interaction partner of RanBP2/Nup358. Interestingly, oocytes had high cytoplasmic levels of RCC1, the Ran nucleotide exchange factor, which is normally excluded from the cytoplasm and enriched on chromatin. RCC1, in turn, may elevate the levels of GTP-bound Ran, which may initiate the RanBP2/Nup358 granule maturation process, in a similar manner to how the Ran-gradient is thought to initiate NPC formation at the nuclear envelope [91]. In unpublished data, the Beck lab has observed that RanBP2/Nup358 can associate with ER-derived membranes that surround the dividing chromosomes in the multinucleated syncytium embryo during mitosis; however, after cellularization, RanBP2/Nup358 is only seen diffusely in mitotic cells, as would be expected. The Beck lab believes that RanBP2/Nup358 helps to seed NPC assembly on these membranes in the syncytium.

RanBP2/Nup358 has been shown to interact with microtubules [92,93] and various cytoskeletal motors such as kinesin-1 and dynein [94–97, 98, 99]. These interactions may help to transport viruses such as HIV between the periphery and the nuclear envelope [82,100] and as discussed above, may move certain biomolecular condensates across the cytoplasm. In addition, these interactions are also required to move the nucleus itself and thus regulate nuclear positioning [97,99]. Nuclear movement is not only important for the function of certain cells but also essential for the cell differentiation of distinct brain progenitor cells, which give rise to the majority of neurons and glia cells of the neocortex [97]. These elongated progenitor cells span the developing cortex and exhibit a characteristic oscillatory movement of the cell nucleus. During apical nuclear migration in the G2 phase of the cell cycle, which is facilitated by dynein motors, the nuclei of these progenitor cells migrate toward the ventricle and the cells can only divide once they reach their destination. Sozanne Solmaz (Binghamton University, USA) discussed the molecular details of how RanBP2/Nup358 interacts with the dynein adaptor Bicaudal D2 (BicD2), which, in turn, recruits the dynein machinery to the nuclear envelope, thus allowing for the migration of the nucleus in neuronal precursor cells. Work from her lab suggests that BicD2 recognizes RanBP2/Nup358 through a ‘cargo recognition alpha-helix’, a structural feature that may stabilize BicD2 in its activated state and promote activation of dynein for processive motility along microtubules [96].

Paulo Ferreira (Duke University, USA) spoke about his lab’s longstanding efforts to understand the role of RanBP2/Nup358 in nuclear/cytoplasmic trafficking in neurons. His group has observed that the selective deletion of RanBP2/Nup358 in mouse motoneurons, results in alterations in the nuclear/cytoplasmic distribution of many substrates of RanBP2/Nup358, including Exportin1, Importin-beta, and HDAC4, and the complete loss of certain proteins, including hnRNPH3 [101]. Ferreira noted that in certain cells, RanBP2/Nup358 localizes to different regions besides the nuclear pore. One of the best examples is in photoreceptor neurons where RanBP2/Nup358 colocalizes with the dense network of mitochondria found in the ellipsoid subcellular compartment [84]. Ferreira thinks that RanBP2/Nup358 may regulate mitochondrial function as the leucine-rich N-terminal domain of RanBP2/Nup358 interacts with Cox11, a chaperone for the Cytochrome C Oxidase, a component of the mitochondrial electron transport chain [102]. Interestingly, Cox11 also inhibits the activity of hexokinase 1, and this inhibition is relieved by the presence of the leucine-rich N-terminal domain. This result may explain why heterozygous knockouts of RanBP2/Nup358 have impaired glucose metabolism, when these mice are challenged with glucose [102]. When RanBP2/Nup358 is deleted from spinal motoneurons in mice, within 12 hours, all RanBP2/Nup358 is lost at the nuclear pore, consistent with it not being a long-lived nuclear pore-associated protein. After 10 days, these mice typically die, and when the spinal motoneurons are imaged, an immunopositive and intranuclear isoform of RanBP2/Nup358, observed in spinal motorneurons to date, relocates to the cytoplasm where it colocalizes with mitochondria [101].

At this point, Ferreira described some more recent work on the RanBP2/Nup358 heterozygous knockout mice. Using a model for age-related macular degeneration (AMD) by exposure of mice to chronic photodamage (a leading risk factor of AMD), his group found that the heterozygous mice were strongly protected against age-dependent oxidative stress-induced apoptosis of photoreceptor neurons [103,104]. This RanBP2/Nup358-environment interaction appears to be partially due to the inhibition of the proteasomal degradation of ubiquitinated proteins. Notably, this age-dependent molecular outcome is recapitulated in mice with loss of the peptidyl-prolyl cis-trans isomerase activity (PPIase) of the cyclophilin domain of RanBP2/Nup358 [105]. Furthermore, a deficit of RanBP2/Nup358 promotes the activation of a neuroprotective factor, phospho-STAT3, which also associates with the cyclophilin domain of RanBP2/Nup358, but independently of this domain’s PPIase activity [101,103]. Given that Shen reported that RanBP2/Nup358 also suppresses STAT1 activity (see above), it is likely that RanBP2/Nup358 regulates many innate immune pathways. By screening inhibitors of the cyclophilin domain’s prolyl isomerase activity, Ferreira’s group have identified small, drug-like compounds that specifically recapitulate the effects of the cyclophilin domain of RanBP2/Nup358 on its substrates, such as the nuclear translocation of STAT3, a decrease of hnRNAPA2B1 and an increase of L/M opsin [106].

The link between RanBP2/Nup358 and mitochondrial function was further explored by Jomon Joseph (National centre for Cell Science, Pune, India). Impaired mitochondrial functions and autophagy have been implicated in many neurological disorders, possibly including ANE [107,108]. Joseph presented his lab work wherein he revealed that RanBP2/Nup358-positive annulate lamellae could often be present at ER-mitochondrial contact sites, which are hubs for regulation of various signaling pathways, intracellular calcium homeostasis, and autophagy [109,110]. Interestingly, the functions of ER-mitochondrial contact sites are dysregulated in several neurodegenerative diseases [111]. He presented evidence suggesting that RanBP2/Nup358 regulates ER-mitochondrial contact sites by suppressing the mTORC2 pathway [112]. Depletion of RanBP2/Nup358 resulted in increased ER-mitochondrial contacts, along with enhanced mTORC2/Akt signaling. The increased contacts between ER and mitochondria upon RanBP2/Nup358 ablation could be rescued by suppressing the mTORC2 pathway, indicating that RanBP2/Nup358 negatively regulates ER-mitochondrial contact sites through restricting mTORC2 signaling. His study also highlighted a role for RanBP2/Nup358 in controlling autophagy that was mediated through the calcium/CaMKK2/AMPK axis. These results unravel new cytoplasmic functions for RanBP2/Nup358 in coordinating cell signaling and calcium-regulated autophagy. Although understanding the physiological relevance of this finding requires further research, the newly discovered roles for RanBP2/Nup358 provide a basis for exploring the possibilities of these processes being altered during ANE development.

## Outstanding questions and future perspectives

There was quite a bit of discussion about the nature of the cytoplasmic foci of RanBP2/Nup358. Some imaging seems to suggest that as an mRNP leaves the nuclear pore, it may actually pick up some RanBP2/Nup358, which would then become part of the mRNP in the cytosol. The idea that cytoplasmic RanBP2/Nup358 foci were derived from the pore-associated fractions is consistent with data from the Ferreira lab [84], data from HIV-infected cells [82], and data from some unpublished imaging of GFP-RanBP2/Nup358 from the Joseph lab, while the idea that these foci contain mRNA is consistent with data from the Beck lab [83]. RanBP2/Nup358 also has numerous RNA-binding regions [24–26], some of which overlap the region responsible for binding to the nuclear pore [24], suggesting that RNA association could potentially release RanBP2/Nup358 from the pore.

Another point that was raised was the effect of the ANE mutations. There was wide agreement that the point mutations associated with ANE are causing some gain of function, but that this is only happening within the right context, be it during viral infection or in a particular cell type. Derek Neilson commented that he has documented individuals who are heterozygous with truncation mutations in the *RANBP2* gene, but they do not suffer from ANE, strongly indicating that ANE-associated mutations have some unique feature in common.

There were also extensive discussions on what exactly is the clinically relevant cell type relevant to ANE. Jefferey Rothstein pointed out that mutations in Nup214, which cause acute infection-induced encephalopathy-9 (IIAE9), begin with a viral injury but cause a progressive neurodegenerative disease [113,114]. These types of diseases may be triggered by cellular stress, but in the long run, cells carrying these mutations cannot cope with the stress and go on to die. Often, the underlying problem with such conditions is a metabolic dysfunction in neurons, and this seems to be common in diseases linked to mutations in nucleoporins. Rothstein also remarked that the composition of the nuclear pore varies between different cell types and encouraged the study of RanBP2/Nup358 with ANE mutations in neuronal tissues. Palazzo suggested that since one of the manifestations of ANE was a cytokine storm, cytokine-expressing cells, such as microglia, may be critical. Arun Venkatesan (John Hopkins University, USA) commented that the pattern of injury seen is compatible with metabolic or mitochondrial disorders and that perhaps the mutations may impact both innate immune cells in the periphery and central nervous tissue. Unlike other neurological diseases associated with mutations in nuclear pore proteins, ANE-associated pathology appears to be limited to viral infections. Moreover, Natalie Arhel pointed to the fact that respiratory viruses, but not other febrile infections, trigger ANE, suggesting that only a subset of infectious agents can provoke this response. Neilson commented that some enteroviruses can also trigger ANE. Matt Elrick (Duke University, USA) pointed out that many enteroviruses are transmitted in a manner similar to respiratory viruses. There was some discussion about whether other related disorders, such as Leigh Syndrome, are also associated with elevated cytokine levels. Derek Neilson remarked that one of the most striking features of the ANE pathology is that upon close inspection of microscopic brain lesions, there is a combination of cell death and capillary proliferation and dilatation, suggesting a problem in cell signaling that is not common to other neuropathologic disorders. To his knowledge, the only similar phenotype that has been reported was in a mouse model where interleukin 6 was over-expressed from a *GFAP* promoter [115]. ANE patients do not typically suffer from hypoxia but rather have cold-like symptoms (cough, fever, and runny nose) than can progress to vomiting and diarrhea before falling into a coma one to two days later. It should be noted that after the conference we were made aware that ANE also shares some similarities with CAR T Cell-Associated Neurotoxicity [116].

The significance of ANE-associated single nucleotide variants in other genes was discussed, in particular, a thermolabile polymorphism in carnitine palmitoyl transferase II [117–119]. Neilson commented that these SNPs were associated with influenza-associated encephalopathy, which has generalized brain swelling and at first appeared to be different from ANE, but more recent work indicates that individuals carrying both the carnitine palmitoyl transferase II thermolabile variant and ANE mutations in RanBP2/Nup358 have stronger recurrence of episodes. Arun Venkatesan commented that although the common final pathology is due to mitochondrial dysfunction, there may be several upstream pathways that can eventually trigger this.

## Data Availability

Data sharing not applicable – no new data generated.
